# Supplementary material for: Biomechanical stress regulates mammalian tooth replacement via the integrin β1‐RUNX2‐Wnt pathway
Source: EMBO J. 2019 Dec 12;39(3):e102374. doi: 10.15252/embj.2019102374 (PMC6996503; doi:10.15252/embj.2019102374)
Supplement: Supplementary file 1 — Appendix [file EMBJ-39-e102374-s001.pdf]

Appendix Files

Biomechanical stress regulates diphyodont tooth replacement via the integrin β1-RUNX2-Wnt pathway

Xiaoshan Wu[1,2], Jinrong Hu[3,4], Guoqing Li[1], Yan Li[1,5], Yang Li[1], Jing Zhang[1], Fu Wang[1,6], Ang Li[1,7], Lei Hu[1], Zhipeng Fan[1], Shouqin Lü[3,4], Gang Ding[1,8], Chunmei Zhang[1], Jinsong Wang[9], Mian Long[3,4] and Songlin Wang[1,9,*]

[1] Beijing Key Laboratory of Tooth Regeneration and Function Reconstruction, Capital Medical University School of Stomatology, Beijing 100050, China.

[2] Department of Oral and Maxillofacial Surgery, Xiangya Hospital, Central South University, Changsha 410008, China.

[3] Center of Biomechanics and Bioengineering, National Microgravity Laboratory, and Beijing Key Laboratory of Engineered Construction and Mechanobiology, Institute of Mechanics, Chinese Academy of Sciences, Beijing 100190, China.

[4] School of Engineering Sciences, University of Chinese Academy of Sciences, Beijing 100049, China.

[5] Fortune Link Triones Scitech Co., Ltd., Beijing 100050, China.

[6] Department of Oral Basic Science, School of Stomatology, Dalian Medical University, Dalian 116044, China.

[7] Key Laboratory of Shaanxi Province for Craniofacial Precision Medicine Research, College of Stomatology, Xi’an Jiaotong University, Xi’an 710004, China.

[8] Department of Stomatology, Yidu Central Hospital, Weifang Medical University, Weifang 262500, China.

[9] Department of Biochemistry and Molecular Biology, Capital Medical University School of Basic Medical Sciences, Beijing 100069, China.

[*]Corresponding author, Tel:+86-010-57099478, E-mail: slwang@ccmu.edu.cn

Table of Contents

Page 3–13: Appendix Supplementary Methods

Page 13–14: Appendix References

Page 15: Appendix Figure S1

Page 16: Appendix Figure S2-S3

Page 17–18: Appendix Figure S4

Page 19: Appendix Figure S5

Page 20–21: Appendix Figure S6

Page 21: Appendix Figure S7

Page 22: Appendix Figure S8

Page 23: Appendix Figure S9

Page 24: Appendix Table S1

Page 25: Appendix Table S2

Appendix Supplementary Methods

In situ hybridization

The procedure of *in situ* hybridization (ISH) consisted of two steps: probe making and section staining. For the first step, RT-PCR for the target gene was performed using mRNA obtained from tooth germ; the primers used for RT-PCR are listed in Appendix Table S1. The DNA was separated using agarose gel electrophoresis. The DNA fragments of the correct size were cut from the agarose gels and were purified. Then, DNA sequencing was performed. The RNA probes were made and labeled with digoxigenin-UTP by *in vitro* transcription with T7 RNA polymerase (10881767001; Roche, Switzerland) and DIG RNA labeling mix solution (11277073910; Roche).

For the second step (staining), the slides were heated and rehydrated first and then treated with proteinase K (1 μg/mL in PBS) at 37°C for 30 min. After refixation with 4% PFA-PBS, the sections were dehydrated in serial alcohol dilutions (25, 50, 75 and 100%) and then left in a biosafety cabinet to air-dry for 1 h. Next, the slides were loaded with diluted probes, covered with plastic cover slips and allowed to hybridize at 70°C overnight. After washing for 3-4 h, the sections were incubated with antibodies (alkaline phosphatase-conjugated anti-digoxigenin, Fab fragments) (11093274910; Roche) overnight. Then the chromogenic signals were detected with the NBT/BCIP substrate (S3771; Promega, Madison, WI). The chromogenic reaction lasted for 12-36 h.

TUNEL assay

The identification of apoptosis was performed with *in situ* apoptosis detection kit (4812-30-K; R&D systems, Minneapolis, MN). Briefly, after rehydration in ethanols, the slides were placed into PBS for 10 min. Then the samples were treated with Proteinase K solution for 15 min, before being rinsed with distilled water. After being incubated with Labeling Reaction Mix at 37 for 1h and then stopped, the samples were washed with PBS and incubated with Strep-Fluorescein Solution. The signal was observed under microscope.

RNAscope assay

For RNAscope assay procedure, the probed region of pig *β-catenin* is listed in Appendix Table S1. The probe was made by Advanced Cell Diagnostics (Newark, CA). The label probe was conjugated to Alexa Fluor 546. The staining protocol was supplied with RNAscope Multiplex Fluorescent Reagent Kit (323100; Advanced Cell Diagnostics). Briefly, after being deparaffinized, the slides were dehydrated with 100% ethanol. The slices were incubated with fresh RNAscope Hydrogen Peroxide (Advanced Cell Diagnostics) for 10 min at RT, and then were washed with distilled water for 4 times. After the target retrieval with RNAscope 1x Target Retrieval Reagent using the steamer, the slides were transferred into 100% ethanol and then dried in 60°C incubator. The RNA RNAscope Protease Plus was added to each section. Then the

slices were incubated at 40°C with probe mix for 2 h at 40°C. After a cascade of signal amplification with RNAscope Multiplex FL v2 AMP 1, 2, and 3, the slides were mounted and the signal was detected.

RNAscope assay is an RNA FISH technique for single molecule detection that can identify the subcellular location of signal (i.e., in the nucleus and cytoplasm) (Arneson et al, 2018; Schulz et al, 2018). We quantified the relative expression level in the nucleus vs. cytoplasm with the following steps. First, the fluorescence image of β-catenin was overlaid with DAPI image to identify the boundary of nucleus. Then, the β-catenin signals within the nucleus boundary were extracted manually through image matting technique with software of Photoshop (Adobe, San Jose, CA). The remaining signals within the cell were regarded as the signals in cytoplasm. The cell numbers were calculated for each figure. Finally, the value of "Integrated Optical Density (IOD) / cell" was calculated for the nucleus or cytoplasm signals with the method which has been described in the main text.

Tests for Young's modulus of specimens

Finite Element Analyses were done to estimate stress inside the mandible (pressure exerted on the inner wall of the mandible). To run the analyses, material properties like Young's modulus and Poisson's ratio were needed. To test Young's modulus (*E*) of specimens, which defines the ability of materials to resist deformation against external forces and is similar to the elastic constant

k of a spring, the bony walls of the mandible slices were cut into small, flat pieces and fixed onto Piuma Chiaro Nanoindenter (Optics11, Amsterdam, The Netherlands) using glue. To be intelligible, the principle of nanoindentation can be explained using Hooke's law ($F = kx$), which describes the relationship between the force exerted (F) and the deformation/elongation (x), and the deformation-resistant parameter k (namely Young's modulus E in mandible) was then obtained based on the F-x (force-deformation) relationship.

In mechanical tests using Optics11, the force exerted on specimens was measured with a force sensor, and the depth of indention (δ)—which resembles x of a spring—was tracked using an interferometer. We chose the Hertz contact model to compute the Young's modulus E—which resembles k of a spring. In total, nine specimens were prepared for the nanoindentation test. For each specimen, more than 10 measuring points were tested with five repetitions for each point, resulting in more than 500 measurements.

Evaluation of varied Poisson's ratio in modeling

The Poisson's ratio is another inherent property of materials, which is to resist volume change against external forces. For most common materials in daily life, the value of the Poisson's ratio ranges from 0-0.5 when assuming isotropy, with "0" referring to infinitely compressible material and "0.5" referring to incompressible material.

Due to the complexity of experimental measurements of the Poisson's

ratio in such diminutive samples, impact of varied Poisson's ratio on the deformation of the mandibles was firstly estimated based on the numerical tests. Different Poisson's ratios (0.15, 0.35 and 0.48) were tested. There was no significant difference upon deformation among the three groups (within one order of magnitude) (Fig EV2F'). Given that the Poisson's ratio of cartilage ranges from 0.15-0.45 (Korhonen et al, 2002; Lai et al, 2015), we used the medium value of 0.35 in all our computational modeling, which is also within the commonly used values in similar simulations (Tomkoria et al, 2004).

Establishment of "Cup model" and calculation of stress value

To simulate the cylinder-like mandible without the "cover", we established the "Cup model" using the finite element analysis software ANSYS 15.0 (ANSYS, Canonsburg, PA). The lingual, labial, anterior, posterior and bottom walls of the mandible were all set as a homogeneous continuum in the model. The most appropriate mesh size was determined for the computation (Fig 2F-H and Fig EV2E-G).

Based on the experimentally measured Young's mudulus and computationally evaluated Poisson's ratio, the stress exerted inside the mandible was calculated as follows (modeling principle). To simplify the interpretation of the modeling, we started with a streamlined one-dimensional uniaxially stretched bar. The length of the bar was recorded as l (geometry), and the length after force applied was recorded as $l + \Delta l$ (Δl is the deformation

of the bar). The deformation per unit length was defined as: $\varepsilon = \Delta l/l$ (extent of deformation or strain). The cross-sectional area of the bar was S (geometry). The force (F) was exerted along the bar, and the force per unit area (namely stress) could be obtained as: $\sigma = F/S$. The Young's modulus was a constant parameter and could be expressed as: $E = \sigma/\varepsilon$. Given the geometry of the object (l, S) and the obtained values of E (Fig EV2D) and ε (Fig 2E), we were able to calculate the force (stress) exerted as: $F = \varepsilon{*}E{*}S$ ($\sigma = \varepsilon{*}E$ or $\varepsilon = \sigma/E$).

Next, we extended the concept to the actual 3-D Cup model. In this 3-D model, the Poisson's ratio should be taken into consideration. Briefly, for a 3-D continuous substance in a state of equilibrium, with the assumption of isotropy and within the regime of small deformation, the strain is defined by the following equation:

$$\varepsilon_{ij} = [\sigma_{ij}(1 + \nu) - \nu\delta_{ij}\sigma_{kk}]/E, \quad (1)$$

wherein (i, j, k) = (x, y, z) denotes the directions of each components of stress (σ) and strain (ε), ν is the Poisson's ratio, and δ_{ij} is Kronecker delta ($\delta_{ij} = 1$ if $I = j$, and $\delta_{ij} = 0$ if $i \neq j$). When the deformation (strain) of the mandible ("cup"), ε_{ij}, is known (experimental measurements) and the two mechanical parameters (the Young's modulus and the Poisson's ratio) are given, the stress on each point of the "cup" (σ_{ij}) can be calculated numerically. Here, σ_{ij} at the boundary of the "cup" is balanced by the pressure exerted on the inner wall of the "cup". When a range of pressure was applied, we got a series of deformation, then the pressure can be assessed by matching the simulated

deformation with that of experimentally measured mandible.

Boundary conditions in modeling and interpretation of simulation results

Boundary conditions in our modeling were used not only for constraining but also for evaluation of the range of pressure exerted on the inner wall of the mandible. For all cases, force was exerted on the inner wall of mandible uniformly and normally, and the bottom of “Cup” was set as fixed boundary to avoid rigid-body motions (Fig 2G).

The outside connective tissue surrounding the mandible restricts the movement of bony walls. To compromise its impact, simplify the Cup model and make full use of experimentally measured mandible deformation as well, we simulated corresponding mandible deformation with a range of pressure exerted on the inner wall under two extreme boundary conditions. For the first condition, the outer surface of the mandible was fixed (radical deformation of the outer surface in the x-z plane was set at 0 in the model (stricter than the real); for the second condition, the outer surface was totally free (looser than the real). The real deformation of the mandible should be between the two conditions, i.e., be smaller than the looser one and bigger than the stricter one.

From the actual surgical experiment on the E60 mandible, we determined that the mean (± SEM) inward displacement of the outer mandible wall was 36.48 ± 4.04 µm, and that of the inner mandible wall was 79.74 ± 6.07 µm. These two values were used to assess the possible range of pressure exerted

on the inner wall of mandible with the model. Fig 2J showed the deformation of mandible walls under a series of stress levels with different Young's moduli. The two values (79.74 and 36.48 µm) were indicated with two upper and lower horizontal grey lines. Take the 0.3 MPa of Young's moduli for example, the dashed green line indicated the result with extreme free outer surface, and solid green line indicated the results with extreme fixed outer surface (those lines were composed of a series of discrete simulations (many data points), however, due to the good linearity of the small deformation system, those points aligned into almost straight lines, thus, points were omitted for concise). There was one intercross point between the upper dashed grey line and the dashed green line, and another intercross point between the lower dashed grey line and the solid green line. The actual pressure exerted on inner wall of the mandible under 0.3 MPa of Young's moduli should be between the two extreme boundary conditions (Inequality of constraint: fixed outer surface>physiological>free outer surface; inequality of deformation: fixed outer surface (strict) <physiological (36.48-79.74 µm)< free outer surface (looser)). More specifically, the calculated maximum pressure should be lower than 13.6 kPa, because the displacement of 36.48 µm from the solid green line (fixed outer surface) should be lower than the lower horizontal grey line (y = 36.48) based on the inequality of deformation. On the other hand, the calculated minimum pressure should be higher than 7.7 kPa, because the displacement of 79.94 µm from the dashed green line (free outer surface) should be higher

than the higher horizontal grey line (y = 79.74) based on the inequality of deformation. As a result, the possible range of 7.7-13.6 kPa was concluded with the 0.3 MPa of Young’s moduli. With the same method, the pressure range of 3-20 kPa was concluded with 0.1-0.6 MPa of Young’s moduli (Fig 2J).

Evaluation of computational results

In the Cup model, we used the maximum deformation of the mandible walls to evaluate the pressure exerted on the inner wall. As a result, the range of the biomechanical stress inside the mandible was relatively large (3-20 kPa) (Fig 2J). Also, in the experiments for testing the Young’s modulus (Fig EV2D), most measurements fell between 0.2-0.3 MPa, with a peak value of 0.23 MPa, indicating that the pressure exerted on the inner wall of the mandible would probably be between 5-13.6 kPa.

In addition, we could estimate the pressure range in another way by using the average deformation of the lingual and labial mandible walls. Simulations showed that the pressure exerted on the inner wall of the mandible would range from 3.5-50 kPa (corresponding to the Young’s modulus spanning from 0.1-0.6 MPa), and, more likely, from 7-17 kPa (corresponding to the peak Young’s modulus of 0.2-0.3 MPa). Since the stress range of 3-20 kPa mostly fell into the range of 3.5-50 kPa, the narrower spectrum of 3-20 kPa was used in the experiments.

***In vitro* mandible culture with chemicals or viruses**

To activate the Wnt pathway, LiCl (20 mM; 746460; Sigma-Aldrich, St. Louis, MO) was added to the culture medium; in the control group, the same concentration of NaCl was added. To inhibit the Wnt pathway, recombinant Dickkopf-related protein 1 (Dkk1) (50 ng/mL; 120-30; PeproTech, Rocky Hill, NJ) was added to the culture medium; in the control group, the same concentration of bovine serum albumin (BSA) was added. To observe the dynamics of Wnt translocation, we harvested the mandible samples treated with recombinant Dkk1 protein or with BSA at 0, 3, 6, 12, and 24 h.

To overexpress RUNX2 in the mandible *in vitro*, we designed a *RUNX2* overexpression lentiviral vector (LV-RUNX2). For this, we cloned the complete mRNA sequence encoding for RUNX2 (XM_013977989.1) into a lentiviral vector (pLenti-Puro-CMV; Vigene Biosciences, Rockville, MD). To label the infected cells, Myc tag was cloned into this vector and a control lentiviral vector. To knock down RUNX2 in the mandible *in vitro*, a *RUNX2* knockdown lentiviral vector was designed: short-hairpin RNA (shRNA) against RUNX2 (5'-GAGAAGGGAAACCUGUGAATT-3') was cloned into lentiviral vector LV3 (GenePharma). Scramble shRNA (5'-TTCTCCGAACGTGTCACGT-3') was cloned into LV3 lentiviral vector as the control. For both the overexpression and knockdown groups, the lentiviral vector (1×10^7 IU/mL) was mixed with hexadimethrine bromide (5 μg/mL) and added to the culture medium. Control viral vector was added to the media of the control groups. The transfection

started at the beginning of the explantation and lasted for 2 days before the samples were harvested.

Western blot of nuclear protein

For Western blotting of nuclear protein, we harvested nuclear protein as follows. DFCs cultured with or without force, with or without *RUNX2* overexpression, and with or without *RUNX2* knockdown were harvested at 70-90% confluence. After centrifuging at 800 x g for 5 min, the cells were packed and the supernatant was discarded. Then, the Cytosol Extraction Buffer (Nuclear-Cytosol Extraction Kit; Applygen Technologies, Beijing, China) was added to the packed cells. After centrifuging at 1000 x g and 4°C for 5 min, we harvested the pellet, which contained the crude nuclei. After washing the pellet at 1000 x g for 5 min, Nuclear Extraction Buffer was added, and the pellet was incubated on ice for 30 min. After centrifuging at 1000 x g and 4°C for 5 min, the supernatant contained the nuclear protein. Presence of Lamin B1 (stained with anti-lamin B1 mouse monoclonal antibody; C1905; Applygen Technologies) was used as internal control for nuclear protein.

References

Arneson D, Zhang G, Ying Z, Zhuang Y, Byun HR, Ahn IS, Gomez-Pinilla F, Yang X (2018) Single cell molecular alterations reveal target cells and pathways of concussive brain injury. *Nat Commun* 9: 3894

Korhonen RK, Laasanen MS, Toyras J, Rieppo J, Hirvonen J, Helminen HJ, Jurvelin JS (2002) Comparison of the equilibrium response of articular cartilage in unconfined compression, confined compression and indentation. *J Biomech* 35: 903-909

Lai YS, Chen WC, Huang CH, Cheng CK, Chan KK, Chang TK (2015) The effect of graft strength on knee laxity and graft in-situ forces after posterior cruciate ligament reconstruction. *PloS one* 10: e0127293

Schulz D, Zanotelli VRT, Fischer JR, Schapiro D, Engler S, Lun XK, Jackson HW, Bodenmiller B (2018) Simultaneous multiplexed imaging of mRNA and proteins with subcellular resolution in breast cancer tissue samples by mass cytometry. *Cell Syst* 6: 531

Tomkoria S, Patel RV, Mao JJ (2004) Heterogeneous nanomechanical properties of superficial and zonal regions of articular cartilage of the rabbit proximal radius condyle by atomic force microscopy. *Med Eng Phys* 26: 815-822

Appendix Figures

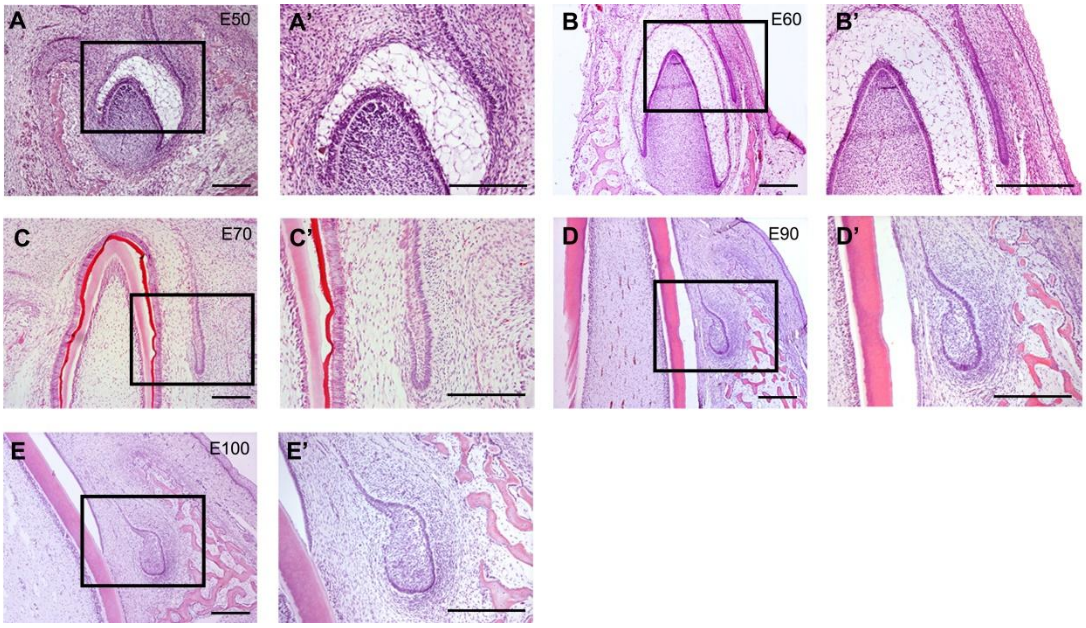

Appendix Figure S1. Morphology of the permanent third incisor from E50 to E100.

A-E and A'-E' The H&E staining showed the morphological changes of the permanent third incisor primordium from embryonic day 50 (E50) to E100, which resembled the permanent canine (PC) primordium. The right figure panels are magnifications of boxed regions in left panels for every group. Scale bars represent 100 µm. n = 3 in all groups.

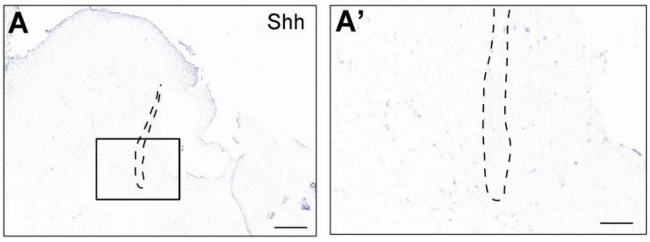

Appendix Figure S2. The expression pattern of *shh* at the primary dental lamina of miniature pig

A-A' In situ hybridization (ISH) expression pattern of *Shh* in the primary dental lamina of the deciduous canine primordium at embryonic day 40 (E40). (A') is the magnification of the boxed region. n = 3. Scale bars represent 100 μm in A and 25 μm in A'.

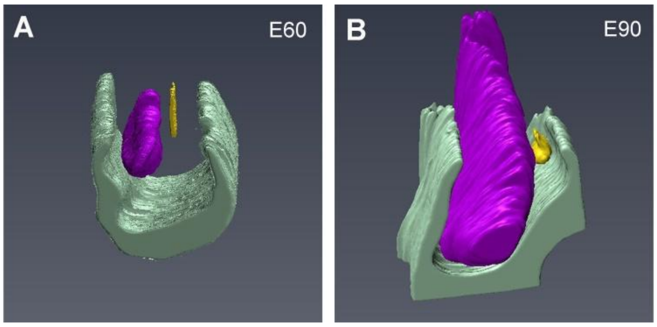

Appendix Figure S3. Three-dimensional reconstructions from serial H&E frontal sections of miniature pig mandible at E60 and E90

A-B Three-dimensional reconstructions from serial H&E frontal sections of miniature pig mandible at E60 and E90; deciduous canine (DC) in *purple*, permanent canine (PC) in *yellow* and alveolar socket in *green*.

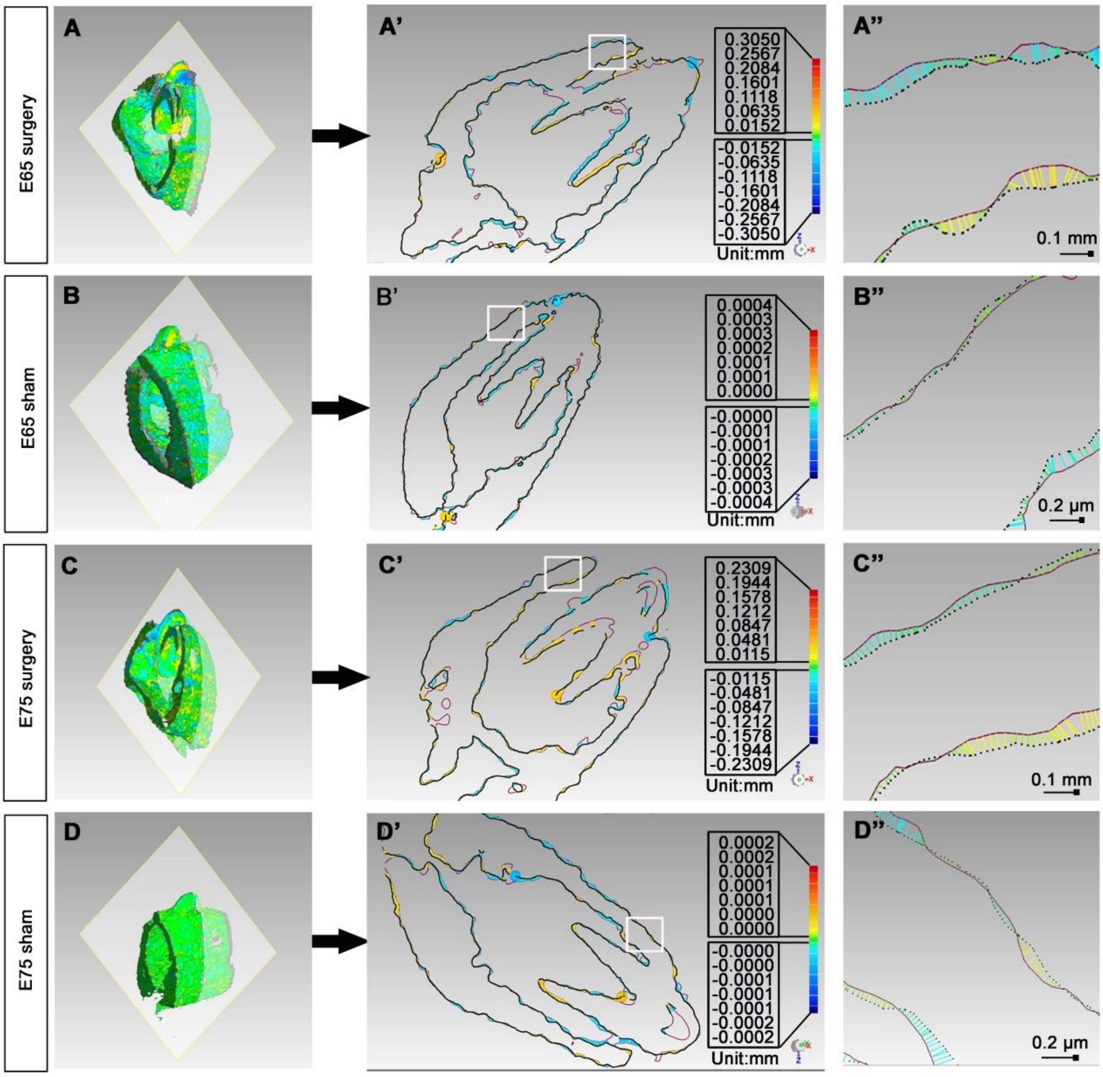

Appendix Figure S4. Three dimensional color map after alignment of E65 and E75 mandible slices before and after surgery indicates the extent of deformation.

A-D Three dimensional color map (*left*) after alignment of E65 and E75 mandible slices before and after (sham) surgery showing comparison results of surface points. Coronal sections through cusp tips (transparent squares, *left*) were selected for 2-D comparison (*middle*); right panels are magnifications of white boxed regions. The solid purple contour and dotted black contour showed the pre-surgery and post-surgery shape,

respectively. The distance between the two contours were the colored line segments showing the distance and direction of the movement. The colored ball in 2-D comparison (*middle*) marked the position of the maximum displacement. n = 3.

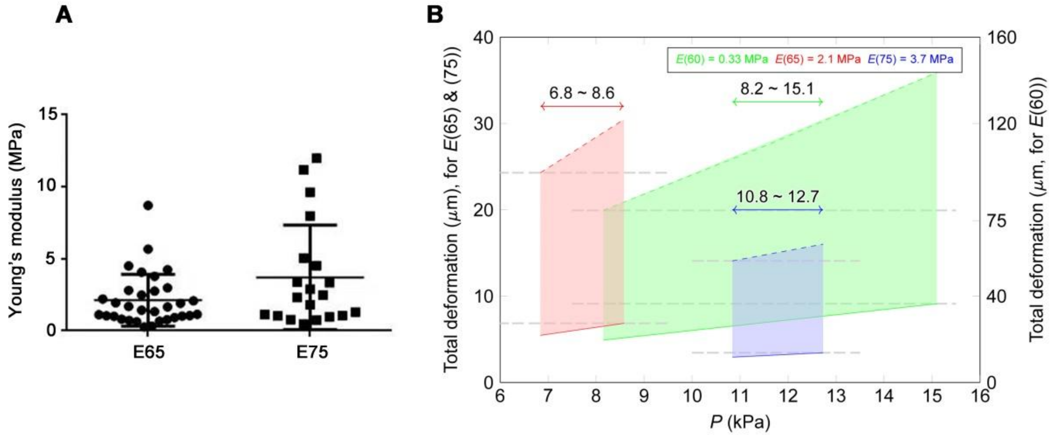

Appendix Figure S5. Deformation of mandible walls under series of stress levels on mandibles

A Scatter diagram of the multiple tests of Young's modulus of the mandible of E65 and E75 stages. $^{*}P < .05$, unpaired two-tailed *t*-test. n = 3.

B Pressure estimation by multiple simulations using mean Young's modulus of E60, E65 and E75 stages. *Gray horizontal lines* indicate upper and lower limit values of mean mandible wall displacement, *dashed colored lines* indicate results with free outer surface, and *solid colored lines* indicate results with fixed outer surface; actual pressure value should be between two extreme boundary conditions. The results showed that probable pressure level ranged from 8.2-15.1 kPa for E60 mandible, 6.8-8.6 kPa for E65 mandible, and 10.8-12.7 kPa for E75 mandible, implying that there may be narrow changes of pressure inside the mandible for the three stages.

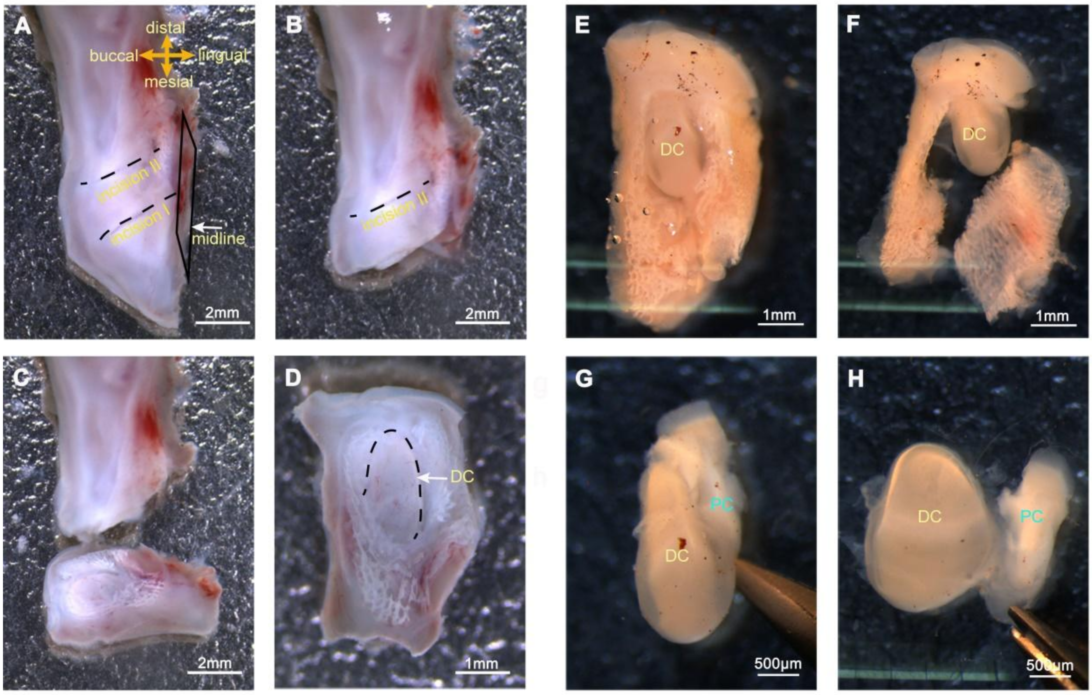

Appendix Figure S6. Micro-dissection of mandible slice and permanent canine germ.

A-D Steps of micro-dissection to obtain the mandible slice containing deciduous canine (DC) and permanent canine (PC). (A) Step 1: Fresh mandible was obtained. The area between two *incision lines* marked the region of canines. (B) Step 2: The first cut was done to remove the incisors. (C) Step 3: The second cut was done to obtain the mandible slice. (D) Step 4: The DC (marked in *dashed line*) could be observed inside mandible.

E-H Steps of micro-dissection to obtain the PC and the surrounding mesenchyme for PT-qPCR. (E) Step 1: The mandible slice was prepared for micro-dissection. (F) Step 2: The lingual and labial bony walls were removed. (G) Step 3: The DC and adjacent PC were harvested. PC was

surrounded by the mesenchymal tissues. (H) Step 4: The tissue containing PC and the surrounding mesenchyme was harvested.

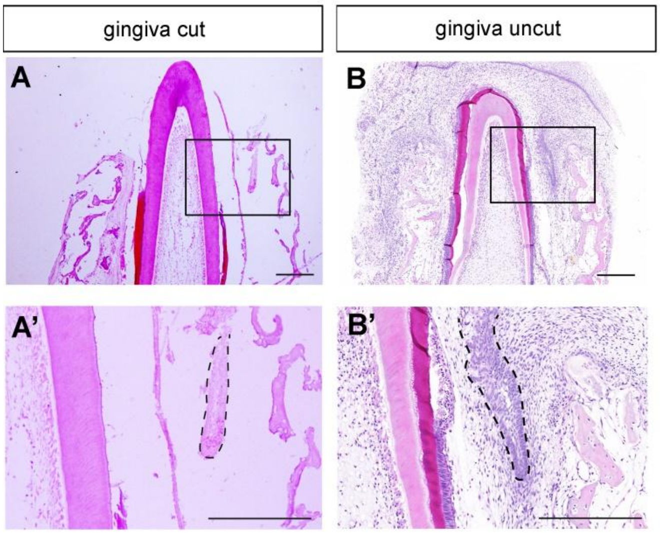

Appendix Figure S7. Morphology of the permanent canine of E60 cultured for 2 days with or without a cut on the top gingiva.

A-B The H&E staining showed the morphological changes of the permanent canine of E60 cultured for 2 days with or without a cut on the top gingiva. The boxed regions in upper panels were magnified in A' and B'. N =3. The scale bars represent 100 μm.

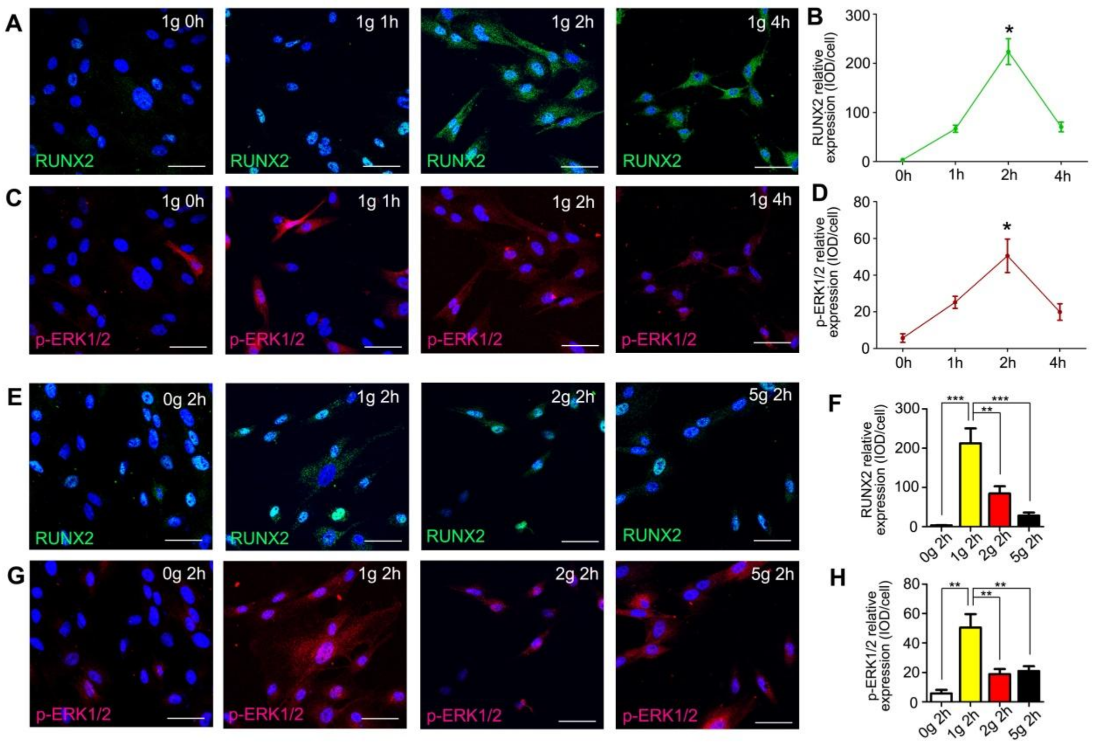

Appendix Figure S8. Immunofluorescence of RUNX2 and p-ERK1/2 upon being compressed with a series of duration or force values.

A-D Immunofluorescence showed the expression levels of RUNX2 and p-ERK1/2 upon loading with 1 g/cm^2 for 0h, 1h, 2h, and 4h. (B,D) The comparisons of the relative expression levels of RUNX2 or p-ERK1/2 of DFCs upon the force of 1 g/cm^2 among the groups of 0h, 1h, 2h, and 4h.

E-H Immunofluorescence showed the expression levels of RUNX2 and p-ERK1/2 upon loading with 0, 1, 2, or 5 g/cm^2 for 2h. (F,H) The comparisons of the relative expression levels of RUNX2 or p-ERK1/2 of DFCs upon loading with 0, 1, 2, or 5 g/cm^2 for 2h.

n = 3 in all experimental groups. The data were presented as mean ± SEM. *P < .05, **P < .01, ***P < .001. Scale bars represent 50 µm.

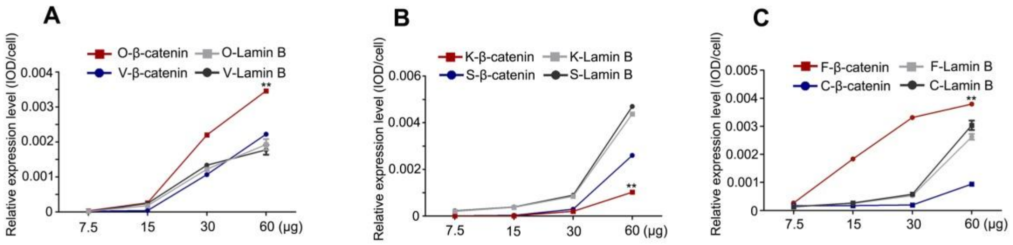

Appendix Figure S9. Comparison of relative expression levels of nuclear β-catenin and Lamin B between groups after semiquantification of western blotting results

A Comparison of relative expression levels of nuclear β-catenin and Lamin B after semiquantification from western blotting between RUNX2 overexpression (O) and control vector (V) groups. The sample was loaded in gradient (7.5, 15, 30, and 60 μg) showing the linear relationship.

B Comparison of relative expression levels of nuclear β-catenin and Lamin B after semiquantification from western blotting between RUNX2 Knockdown (K) and Scramsh (S) groups. The sample was loaded in gradient (7.5, 15, 30, and 60 μg) showing the linear relationship.

C Comparison of relative expression levels of nuclear β-catenin and Lamin B after semiquantification from western blotting between compressed force (F) (1.0 g/cm2) and control (C) (0 g/cm2) groups. The sample was loaded in gradient (7.5, 15, 30, and 60 μg) showing the linear relationship.

n = 3 in all experimental groups. The data were presented as mean ± SEM. $^{**}P < .01$.

Appendix Tables

Appendix Table S1 Primers of genes for *in situ* hybridization

Gene name	Accession no.	Sense primer	Antisense primer	Size of PCR product (bp)
pig Shh	NM_001244513.1	agcagtttatccccaacgtg	ctctctcgtctcgatcac	644
pig Pitx2	NM_001206435.1	acccgtccaagaagaagagg	ctcgagttacacgtgtccct	607
pig Pax9	XM_005666200.2	aacagggccattacgactca	agcagcactgtaggtcatgt	470
pig Sox2	NM_001123197.1	gaacagcccagaccgagtta	agccgttcatgtaggtctgc	554
pig integrin β1	XM_021064065.1	ccccaagtcagcagtaggaa	cacacactcgacacttgcaa	683
pig ERK1	XM_013991188.1	gcgtttgttcttggctcctt	agctccatgtcgaaggtgaa	450
pig RUNX2	XM_005666074.3	aaccacagaaccacaagtgc	cacagagcacaggaagttgg	554
pig β-catenin	XM_021068566.1	ggtccatcagctttccaaaa	ctgaacaagggtcccaagaa	700
pig Sfrp1	XM_003359868.5	caacaagaactgccacatcg	aagtggtggctgaggttgtc	501
pig Sostdc1	XM_003482678.4	aaacctgttccagcacacac	ctgccgtgtgtatctcttgc	402
human integrin β1	NM_033668.2	atgaagggcgtgttggtaga	ggacacaggatcaggttgga	593
human ERK1	NM_001040056.2	aagtacatccactccgccaa	actgtaggtagtttcgggcc	385
human RUNX2	NM_001015051.3	tcctccccaagtagctacct	tgaaatgcttgggaactgcc	457

Appendix Table S2 Primers of genes for RT-PCR

Gene name	Accession no.	Sense primer	Antisense primer
TGFB1	NM_214015.2	caaggtcctggctctgtaca	gaacgcacgatcatgttgga
NFKB1	NM_001048232.1	ggacgaggatggagaggatg	tcatgtctccttgtgcgagt
EGR1	XM_003123974.6	cttcgcctgtgacatctgtg	gggtaggaagggagagagga
RUNX2	XM_005666074.3	ggcagttcccaagcatttca	caggtaggtgtggtagtggg
integrin β1	NM_213968.1	gcaatgggacgtttgagtgt	cgcagacacactctccattg
integrin β3	NM_214002.1	cgacttctcctgtgtccact	ttgcagtagaagccagtcca
integrin αV	NM_001083932.1	agactgaggaagacgttggg	gcagttcattggcccatcaa
integrin α2	NM_001244272.2	ttttgcttcctggtccatgc	aactgacagatgcgcgattc
Gapdh	NM_001206359.1	tcctgggctacactgaggac	ccctgttgctgtagccaaat
