# Supplementary material for: Biomechanical stress regulates mammalian tooth replacement via the integrin β1‐RUNX2‐Wnt pathway
Source: EMBO J. 2019 Dec 12;39(3):e102374. doi: 10.15252/embj.2019102374 (PMC6996503; doi:10.15252/embj.2019102374)
Supplement: Supplementary file 2 — Expanded View Figures PDF [file EMBJ-39-e102374-s002.pdf]

Expanded View Figures

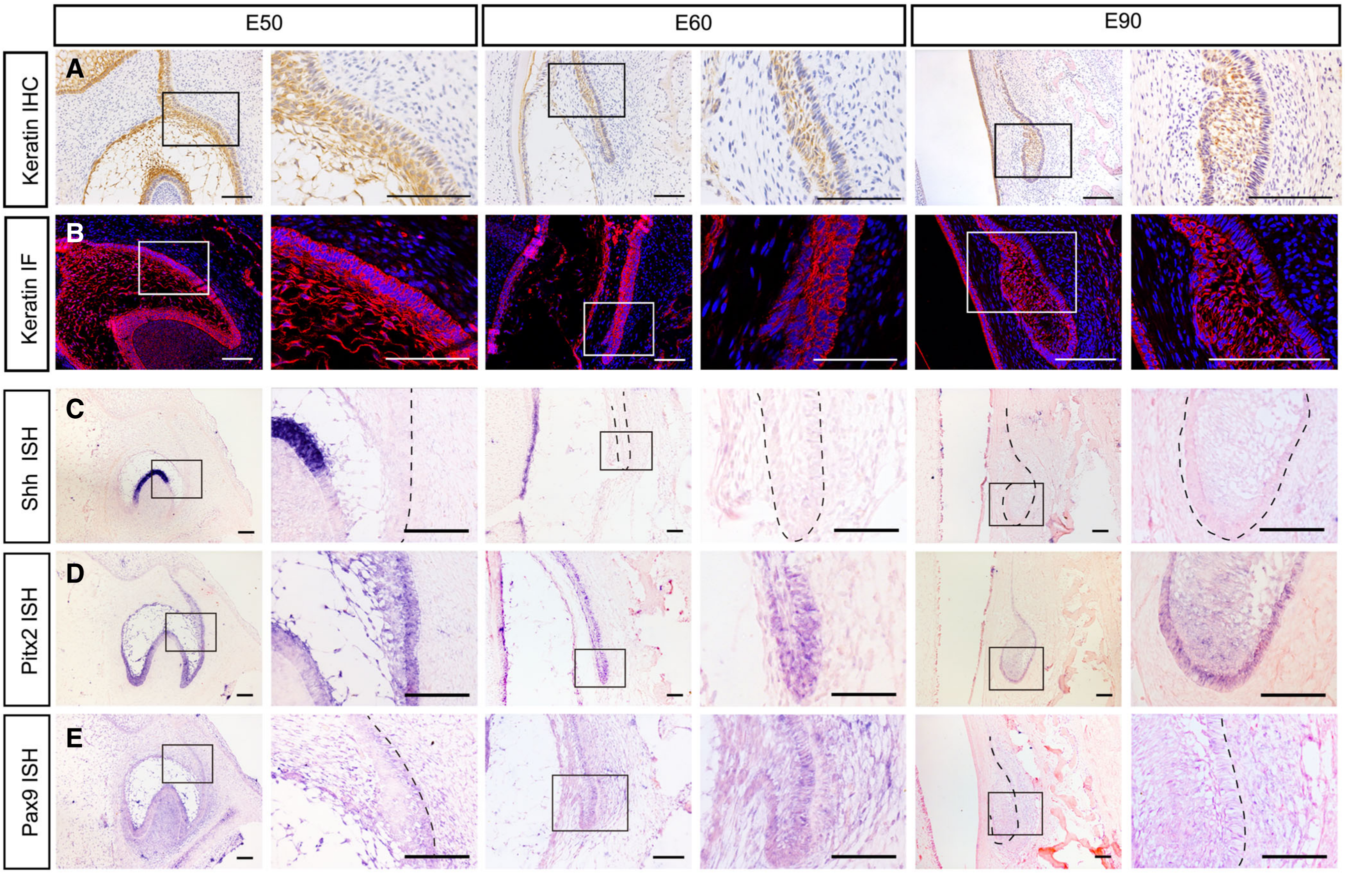

Figure EV1. Immunostaining and *in situ* hybridization (ISH) of the PC primordium from E50 to E90.

A Immunohistochemistry (IHC) of pan-cytokeratin from embryonic days 50 (E50) to E90 showing the dual layers of the epithelium in dental lamina and enamel organ.

B Immunofluorescence (IF) of pan-cytokeratin from E50 to E90 showing a similar pattern.

C–E ISH of *Shh*, *Pitx2*, and *Pax9* during the initiation stage from E50 to E90. Dashed lines mark the position of the SDL or PC.

Data information: $n = 3$ for all panels. Right figure panels are magnifications of the boxed regions in corresponding left panels. Scale bar = 100 µm.

 © 2019 The Authors

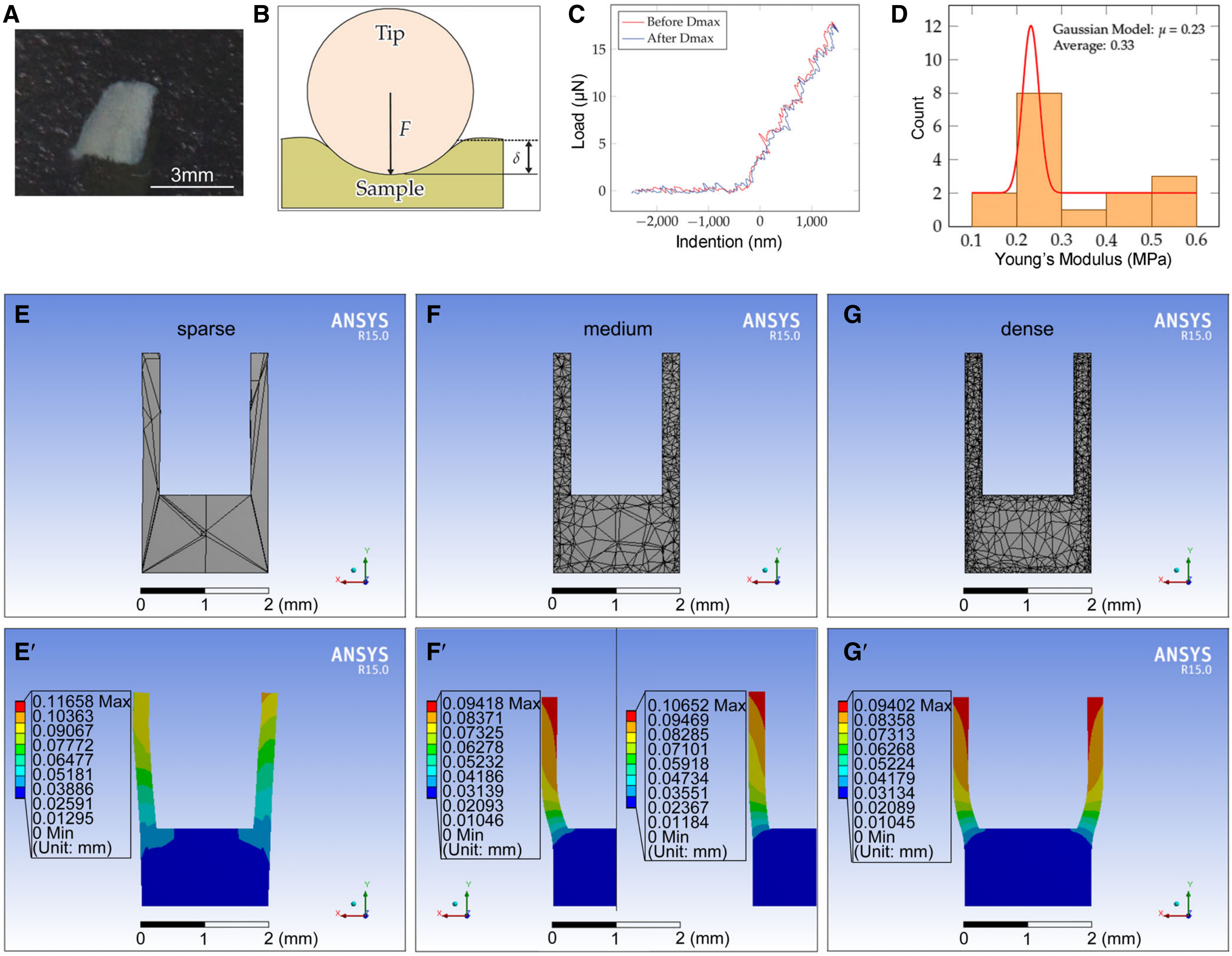

Figure EV2. Mechanical test and establishment of the cup model.

A–D Testing Young's moduli of the samples. (A) Mandible bony piece (mandible side wall piece) prepared for the test (0.2–0.3 mm in thickness). (B) Diagram of the testing principle of nanoindentation for Young's modulus determination using the Piuma Chiaro Nanoindenter. The indenter (tip) applies force onto the sample (mandible bony piece in A) and records the force applied and the indentation depth (sample deformation). (C) Normal force spectrum from which valid experimental data were extracted. The red line signifies that the probe was engaging before reaching max deformation (Dmax); blue line indicates that the probe retreated after reaching Dmax. (D) Distribution of the typically measured Young's moduli of the samples. Red line indicates the fitting results of the Gaussian model.

E–G Gradient densities of the mesh (sparse, medium, and dense) were tested to determine the optimal mesh density of the cup for the computation. (E, E') The deformation is not symmetrical although it is clear in the sparse group. (F, F' left, G, and G') The medium and dense groups show similar deformation after exerting a force on the inner wall. (F') Series of Poisson's ratios, including 0.15 (not shown), 0.35 (left), and 0.48 (right), were tested. Dmax values of the three cases were 1, 1.21, and 1.37 after normalizing the data.

© 2019 The Authors

© 2019 The Authors

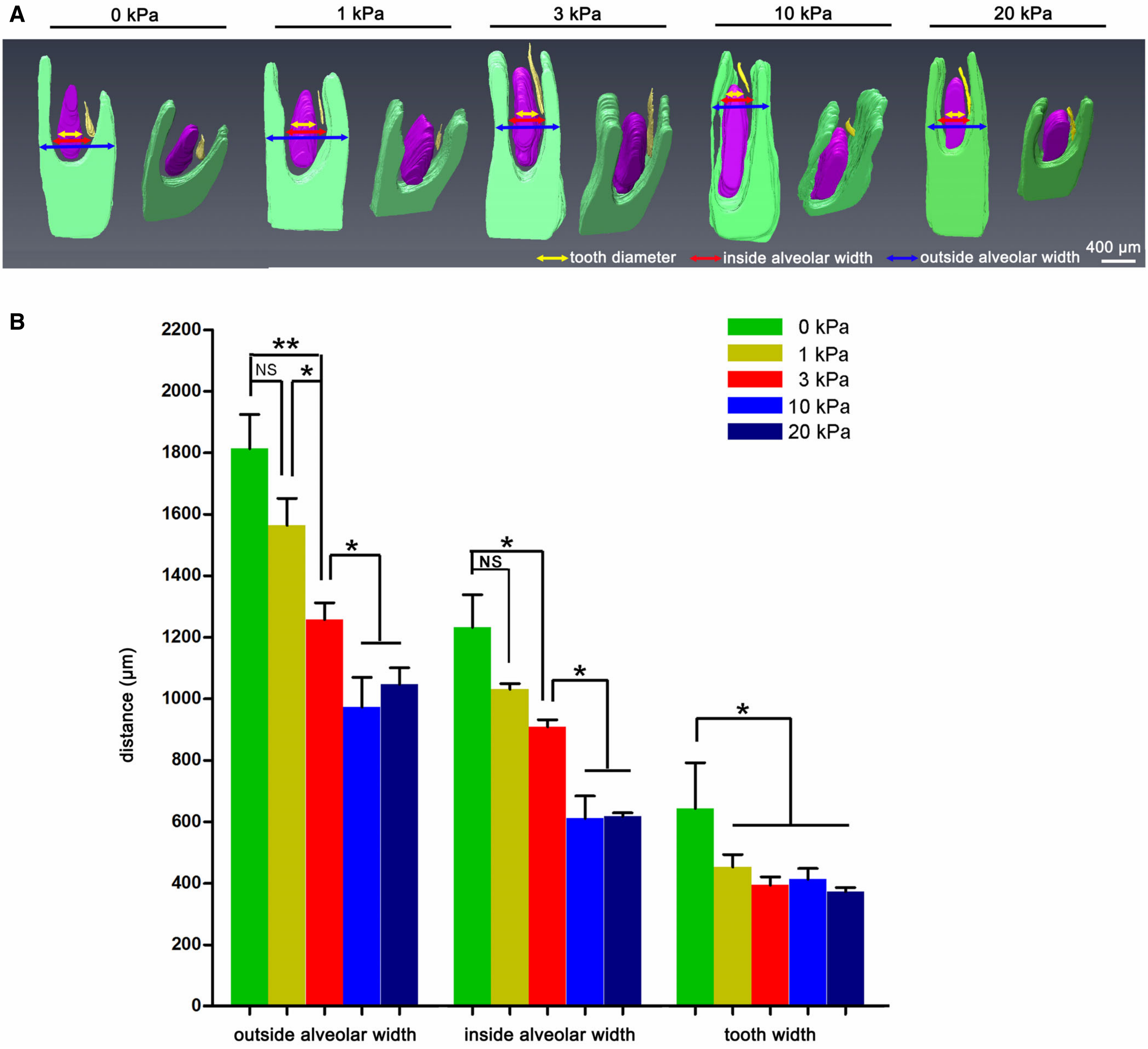

Figure EV3. Morphological comparisons of cultured mandible slices subjected to different pressures.

A Morphological comparison of 3D reconstructions from a series of H&E sections of the mandible cultured under 0, 1, 3, 10, and 20 kPa. The deciduous canine (DC) is colored purple, the permanent canine (PC) germ is colored yellow, and the mandible is colored green. Arrows in yellow, red, and blue indicate the DC, inside alveolar, and outside alveolar widths, respectively.

B Quantitative comparison of the three parameters among five groups. $n = 3$ for all groups. Data represent the mean ± SEM. One-way ANOVA (Newman–Keuls test for post hoc comparisons between two groups), $*P < 0.05$; $**P < 0.01$; NS, not significant.

 © 2019 The Authors

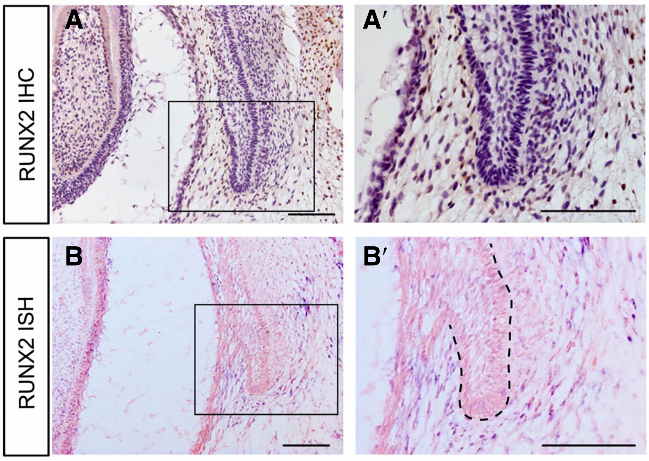

Figure EV4. Expression patterns of RUNX2 in the third deciduous incisor during resting stage of E60.

A, B The expression pattern of RUNX2 in the permanent third incisor at E60 shows similarities with that of the PC via IHC and ISH assays. The boxed regions are magnified in (A' and B'). Dashed lines mark the epithelium of PC. n = 3. Scale bars = 100 µm.

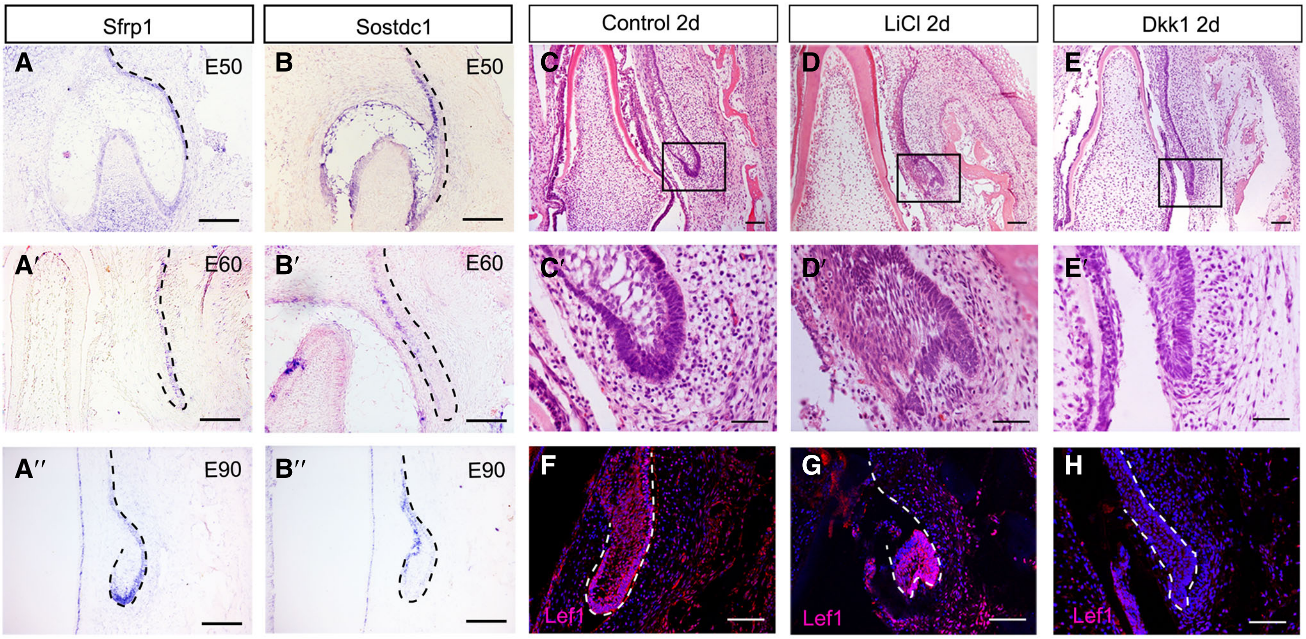

Figure EV5. The Wnt/β-catenin signaling pathway regulates permanent canine initiation.

A–A" Wnt inhibitor *Sfrp1* expressed in the outside layer of the PC SDL from E50 to E90.

B–B" Wnt inhibitor *Sostdc1* expressed in the inside layer of the SDL from E50 to E90, complementary to the pattern of *Sfrp1*.

C–E Morphological comparisons between the control, LiCl, and Dkk1 groups showing initiation of PC in the control and LiCl groups, but inhibition in the Dkk1 group. The boxed regions are magnified in the lower panels (C', D', and E').

F–H IF of Lef1 showing strong positive signals in the enlarged PC epithelium and surrounding mesenchyme in LiCl and control groups, and a weak signal in the Dkk1 group.

Data information: n = 3 for all experimental groups. Dashed lines mark the successional dental lamina or epithelium of PC. Scale bars = 100 µm (A–A" and B–B"), 50 µm (C–H), and 25 µm (C'–E').

© 2019 The Authors
