# Supplementary figures and images for: Biomechanical stress regulates mammalian tooth replacement via the integrin β1‐RUNX2‐Wnt pathway
Source: EMBO J. 2019 Dec 12;39(3):e102374. doi: 10.15252/embj.2019102374 (PMC6996503; doi:10.15252/embj.2019102374)

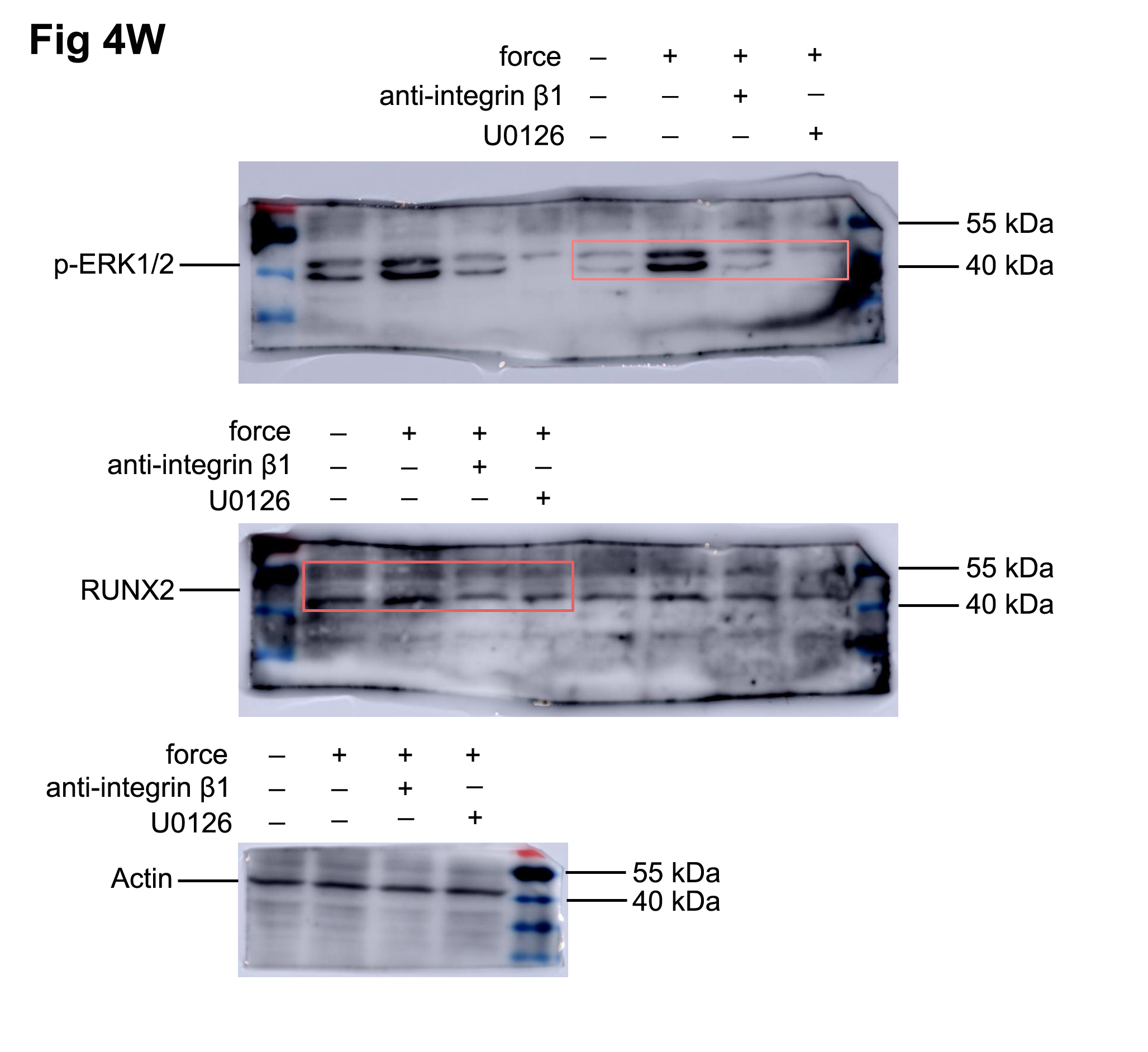

Supplement: Supplementary file 4 — Source Data for Figure 4 [file EMBJ-39-e102374-s003.jpg]

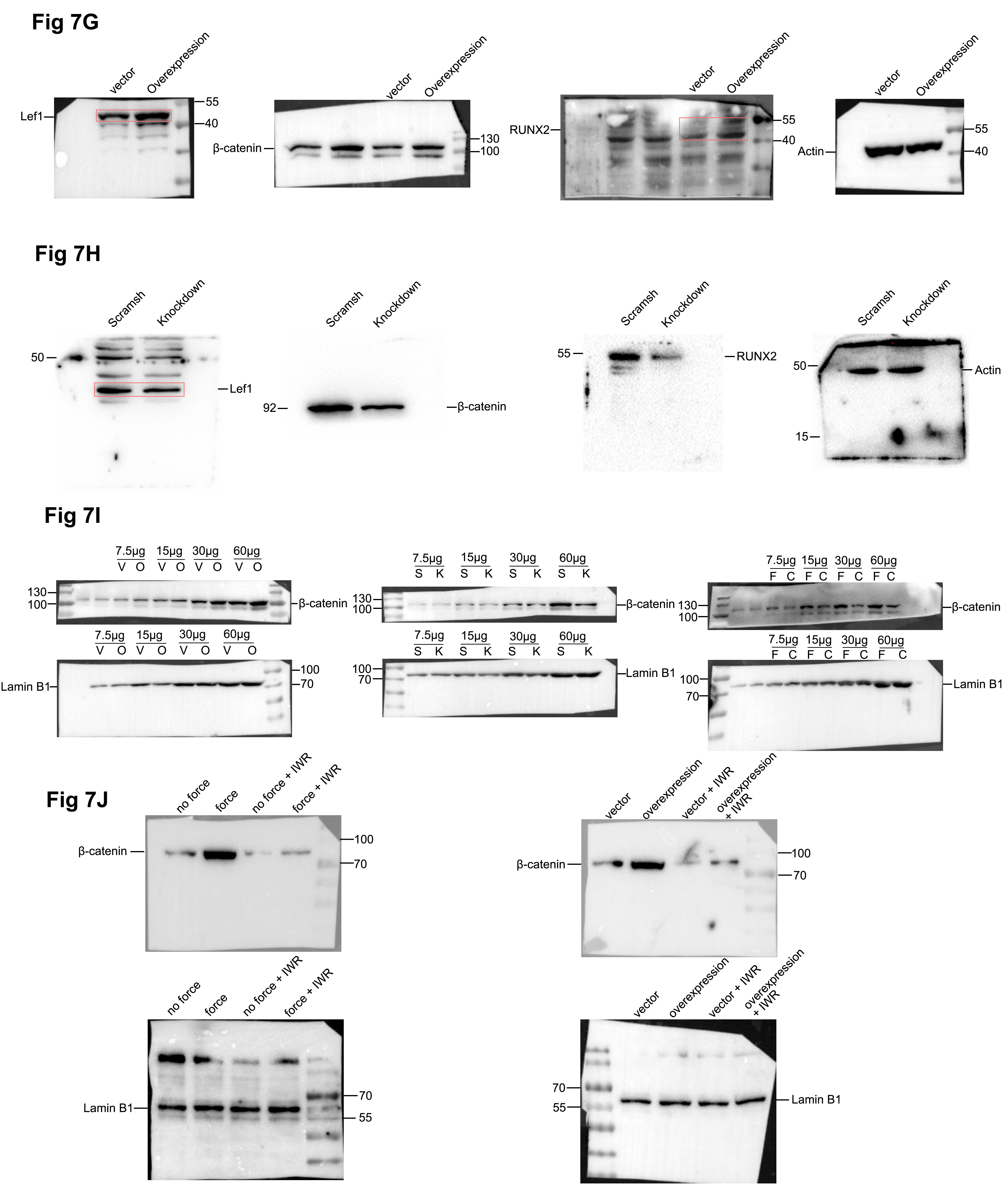

Supplement: Supplementary file 5 — Source Data for Figure 7 [file EMBJ-39-e102374-s004.jpg]
